# Supplementary material for: GDF11 Regulates PC12 Neural Stem Cells via ALK5-Dependent PI3K-Akt Signaling Pathway
Source: Int J Mol Sci. 2022 Oct 14;23(20):12279. doi: 10.3390/ijms232012279 (PMC9602726; doi:10.3390/ijms232012279)
Supplement: Supplementary file 1 [file ijms-23-12279-s001.zip › File S1.pdf]

The uncropped blots of Figure 1D

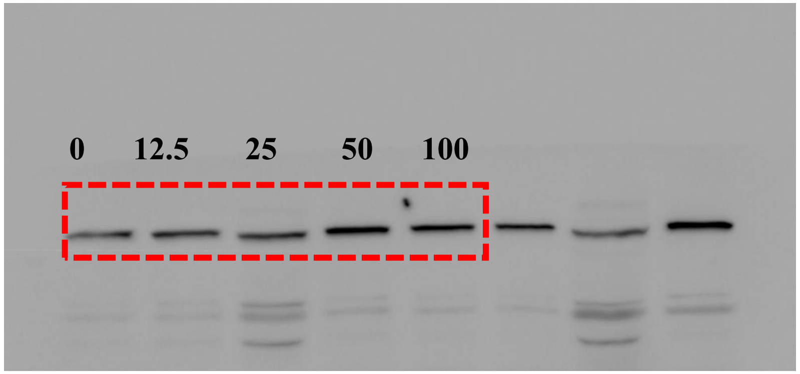

GFAP for Figure 1D

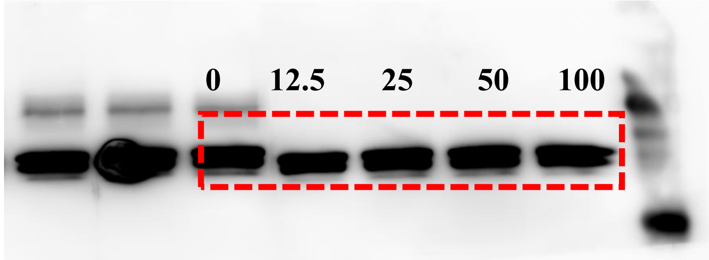

β-actin for Figure 1D

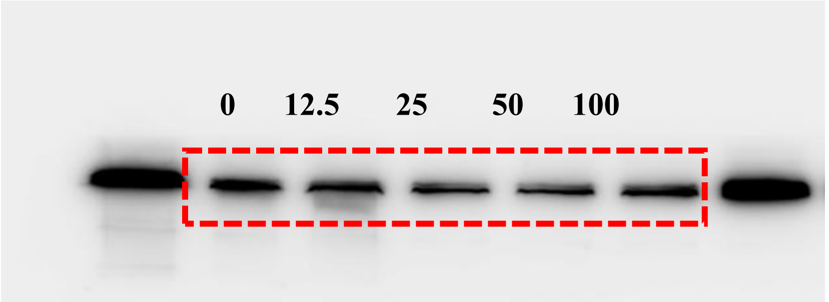

Nestin for Figure 1D

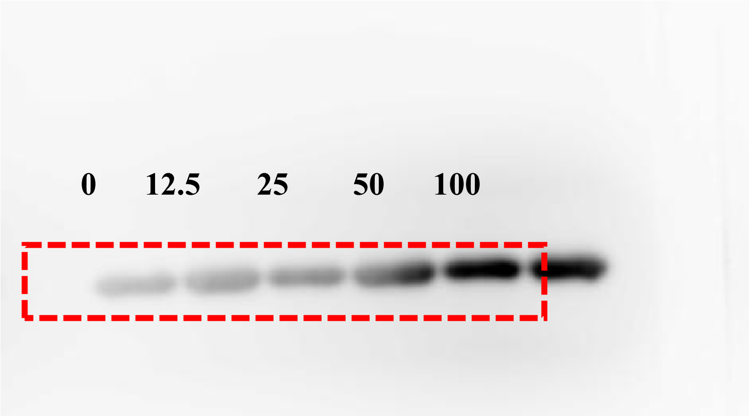

tubulin for Figure 1D

The uncropped blots of Figure 7A

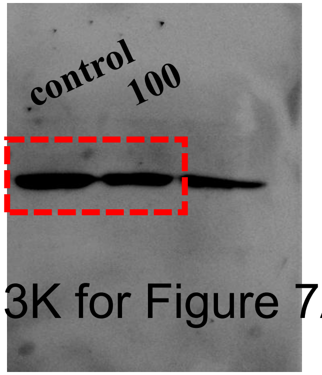

PI3K for Figure 7A

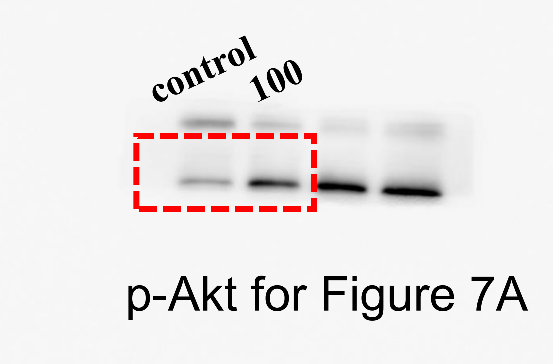

p-Akt for Figure 7A

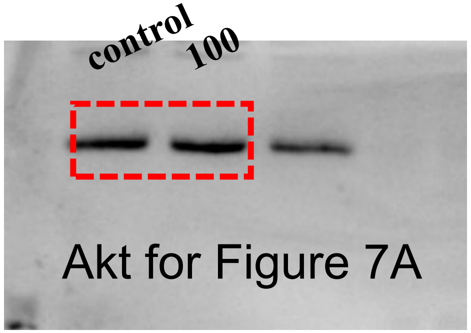

Akt for Figure 7A

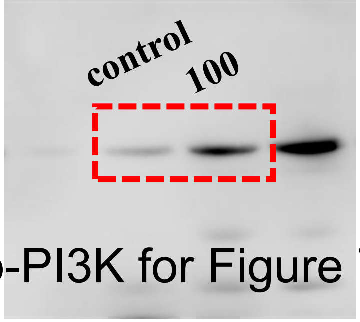

p-PI3K for Figure 7A

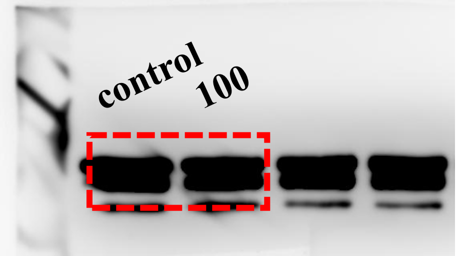

GAPDH for Figure 7A

The uncropped blots of Figure 7D

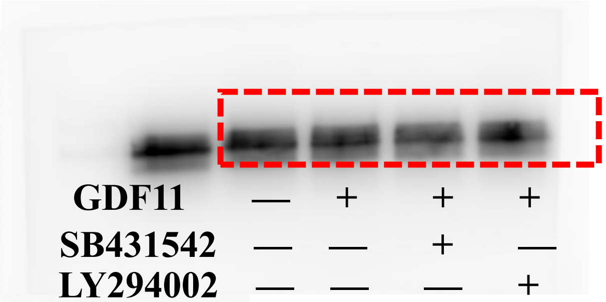

Akt for Figure 7D

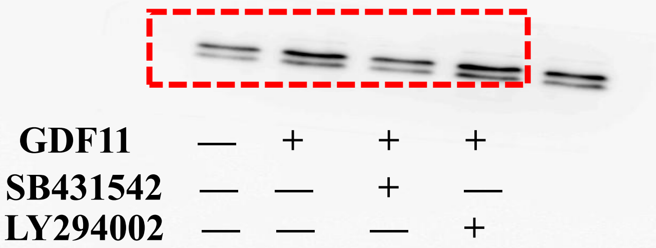

p-Smad2/3 for Figure 7D

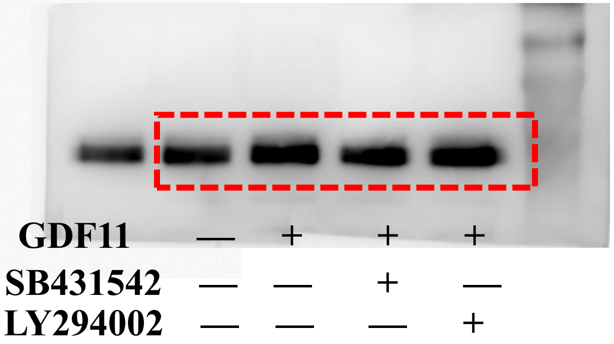

GAPDH for Figure 7D

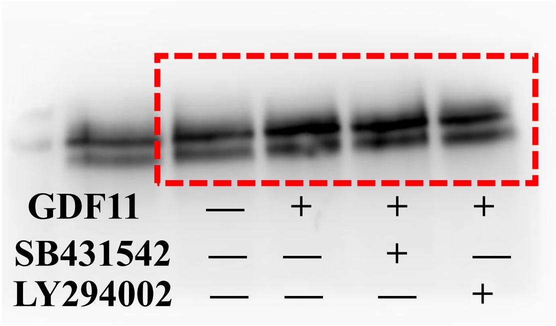

Smad2/3 for Figure 7D

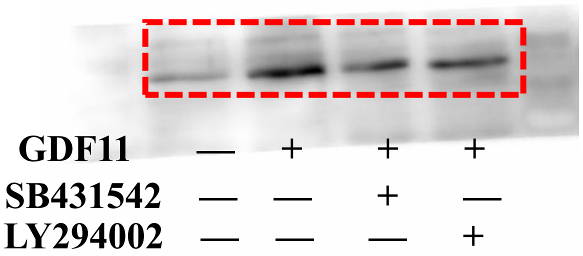

p-Akt for Figure 7D

The uncropped blots of Figure 7F

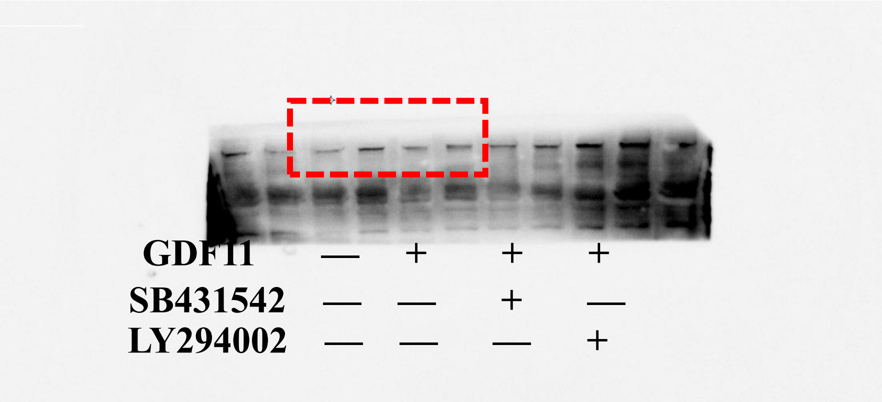

Cleaved Caspase-3 for Figure7F

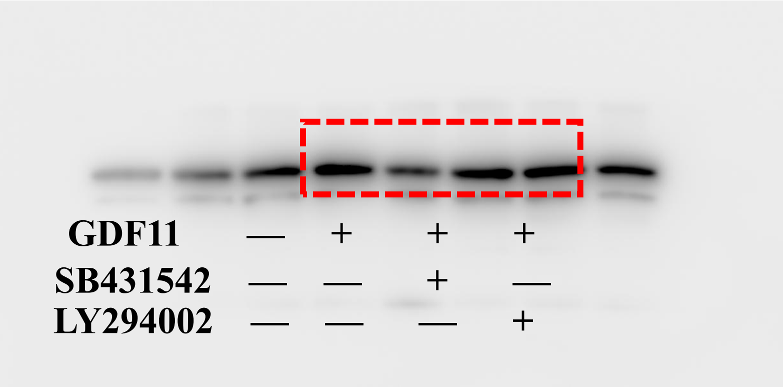

BCL-2 for Figure7F

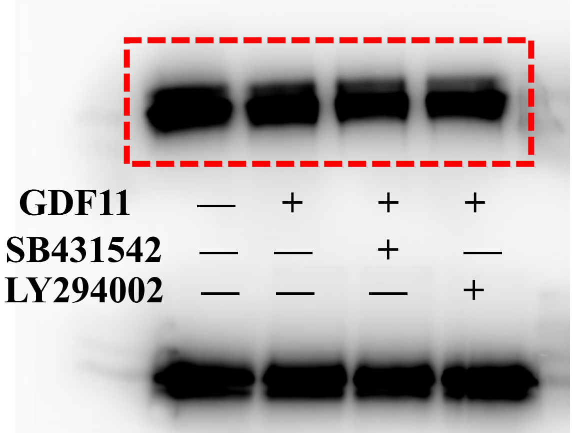

β-actin for Figure7F

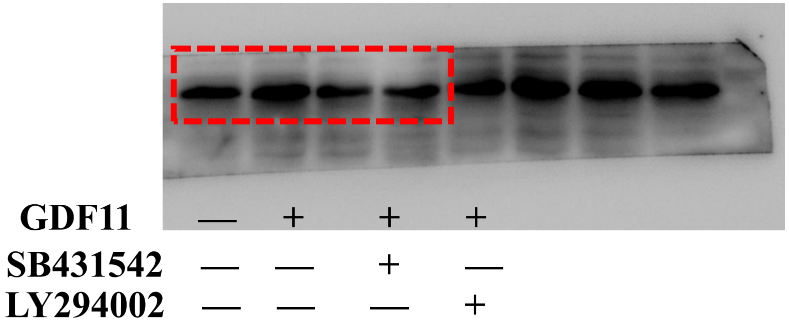

Bax for Figure7F
