# Supplementary material for: GDF11 Regulates PC12 Neural Stem Cells via ALK5-Dependent PI3K-Akt Signaling Pathway
Source: Int J Mol Sci. 2022 Oct 14;23(20):12279. doi: 10.3390/ijms232012279 (PMC9602726; doi:10.3390/ijms232012279)
Supplement: Supplementary file 1 [file ijms-23-12279-s001.zip › Table S1 and suppl. figures.pdf]

Table S1. Primer sequence for qRT-PCR

primers	Forward (5'-3')	Reverse(5'-3')
GAPDH	GGCATTGCTCTCAATGACAA	TGTGAGGGAGATGCTCAGTG
Nestin	GGAGGGCAGAGAAGACAGTG	TGACATCCTGGACCTTGACA
β-actin	GGCTGTATTCCCCTCCATCG	TAAAGACCTCTATGCCAACACAGT
GFAP	CACGAACGAGTCCCTAGAGC	TCACATCACCACGTCCTTGT
βIII-tubulin	GAATGACCTGGTGTCCGAGT	CAGAGCCAAGTGGACTCACA
Cyclin D2	CTGTGCGCTACCGACTTCAA	GCAGAGCTTCGATTTGCTCC
Rasal2	ATGGAGCTGTCTCCGTCGT	GCCTTTTACATCGAACACCCG
Cyclin D1	CCCTGACACCAATCTCCTCA	TCTTCTTCAAGGGCTCCAGG
Cdkn1B	TCAAACGTGAGAGTGTCTAACG	CCGGGCCGAAGAGATTTCTG
Trp53bp2	AGTAAAGGCTCTAAAGCTCACCC	GTAAGAGGTCGGCATTGGAAG
Ccnyl1	GAGAGGCTCCTAACTTATGCTGA	CCTGGTCATCCCAAACCTTG
Cyclin B1	AAGGTGCCTGTGTGTGAACC	GTCAGCCCCATCATCTGCG
Cyclin A2	GCCTTCACCATTCATGTGGAT	TTGCTGCGGGTAAAGAGACAG

Figure S1

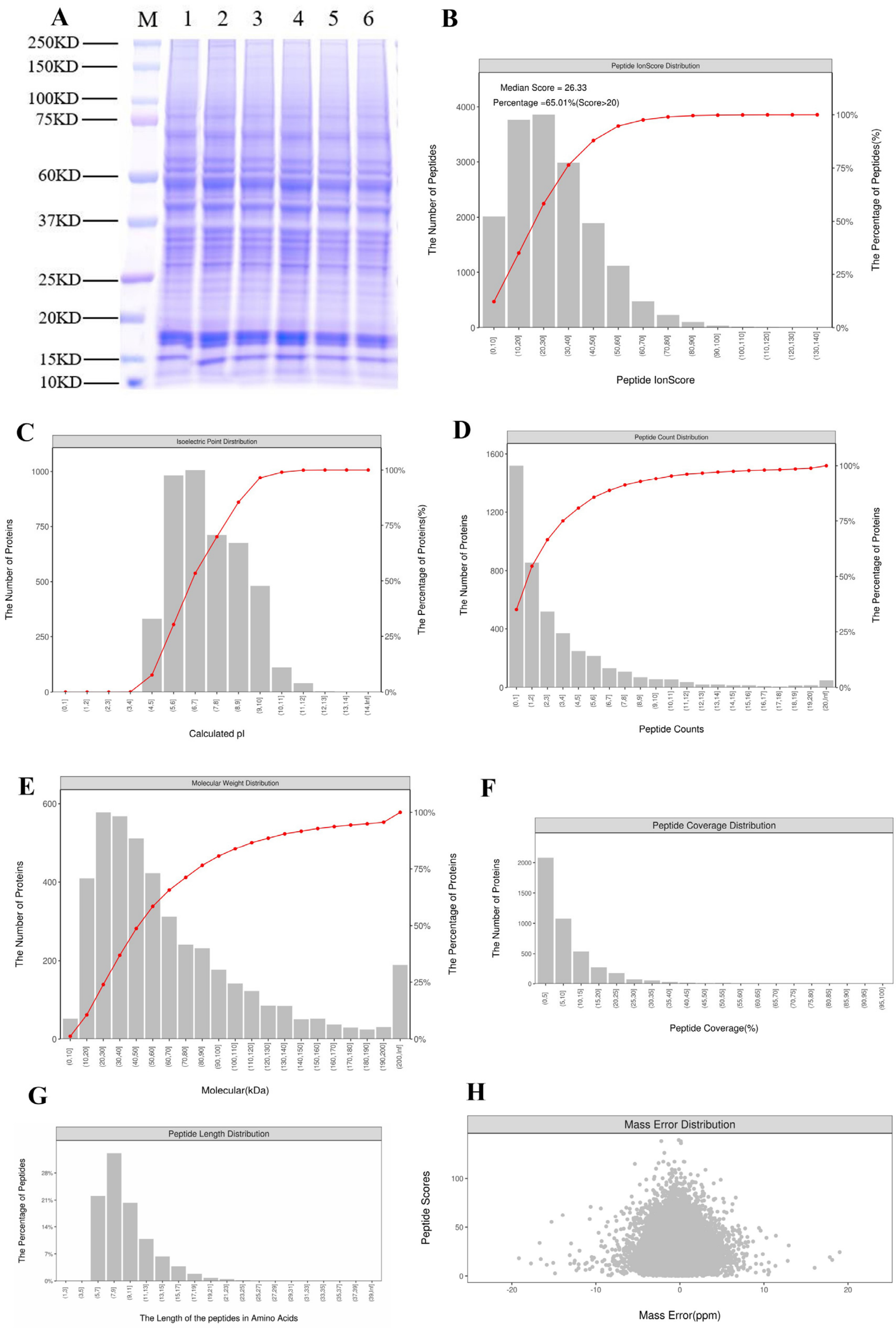
A
M 1 2 3 4 5 6
250KD
150KD
100KD
75KD
60KD
37KD
25KD
20KD
15KD
10KD
B
Peptide IonScore Distribution
Median Score = 26.33
Percentage =65.01%(Score>20)
The Number of Peptides
The Percentage of Peptides(%)
Peptide IonScore
C
Isoelectric Point Distribution
The Number of Proteins
The Percentage of Proteins(%)
Calculated pI
D
Peptide Count Distribution
The Number of Proteins
The Percentage of Proteins
Peptide Counts
E
Molecular Weight Distribution
The Number of Proteins
The Percentage of Proteins
Molecular(kDa)
F
Peptide Coverage Distribution
The Number of Proteins
Peptide Coverage(%)
G
Peptide Length Distribution
The Percentage of Peptides
The Length of the peptides in Amino Acids
H
Mass Error Distribution
Peptide Scores
Mass Error(ppm)

Figure S2

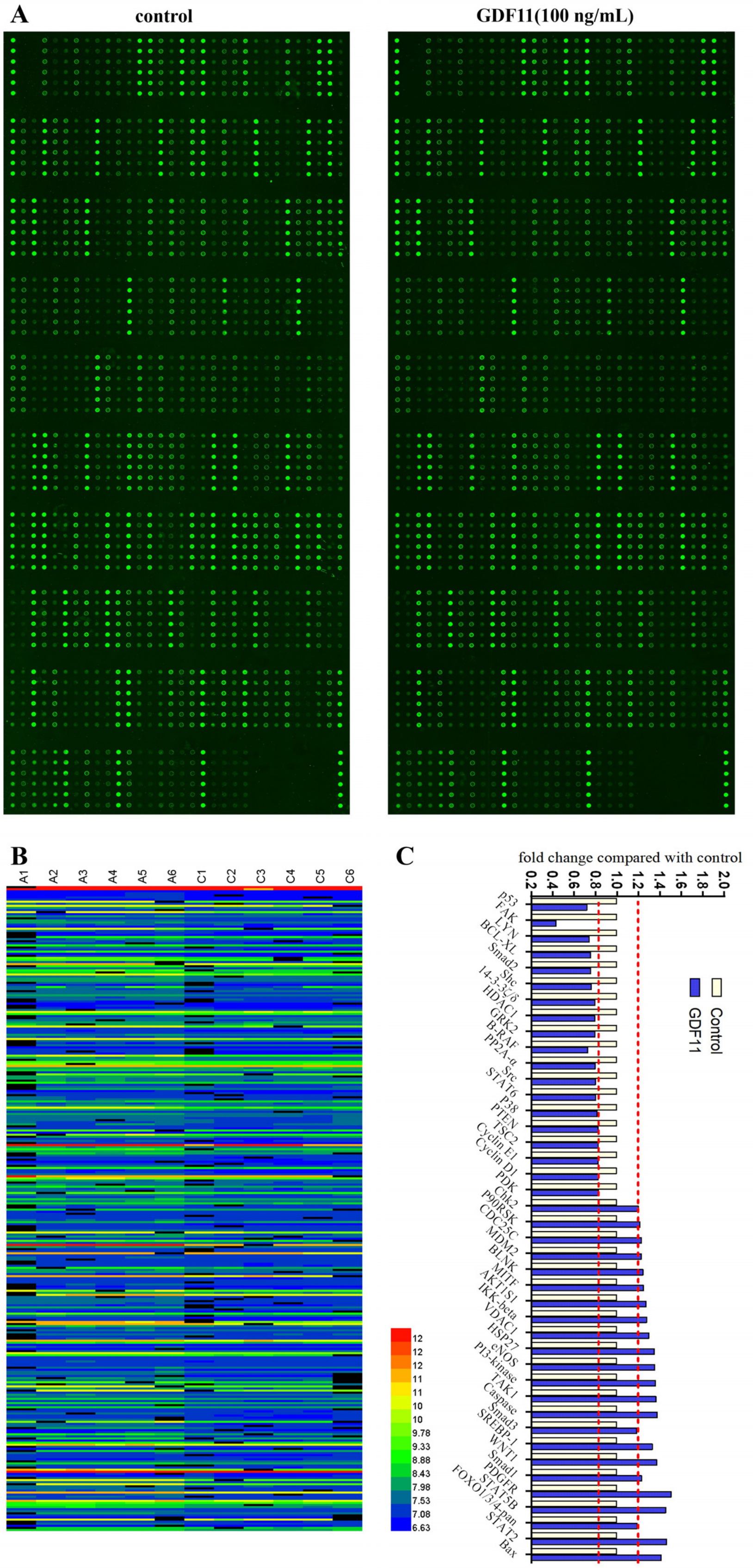
A
control
GDF11(100 ng/mL)
B
A1
A2
A3
A4
A5
A6
C1
C2
C3
C4
C5
C6
12
12
12
11
11
10
10
9.78
9.33
8.88
8.43
7.98
7.53
7.08
6.63
C
fold change compared with control
0.2
0.4
0.6
0.8
1.0
1.2
1.4
1.6
1.8
2.0
Control
GDF11
p53
FAK
LYN
BCL-XL
Smad2
Shc
HDAC1
GRK2
B-RAF
PP2A-α
Src
STAT6
P38
PTEN
TSC2
Cyclin E1
Cyclin D1
PDK
Chk2
P90RSK
CDC25C
MDM2
BLNK
MITF
AKT1S1
IKK-beta
VDAC1
HSP27
eNOS
PI3-kinase
TAK1
Caspase
Smad3
SREBP-1
WNT1
Smad1
PDGFR
STAT5B
FOXO1/3/4-pan
STAT2
Bax

Figure S3

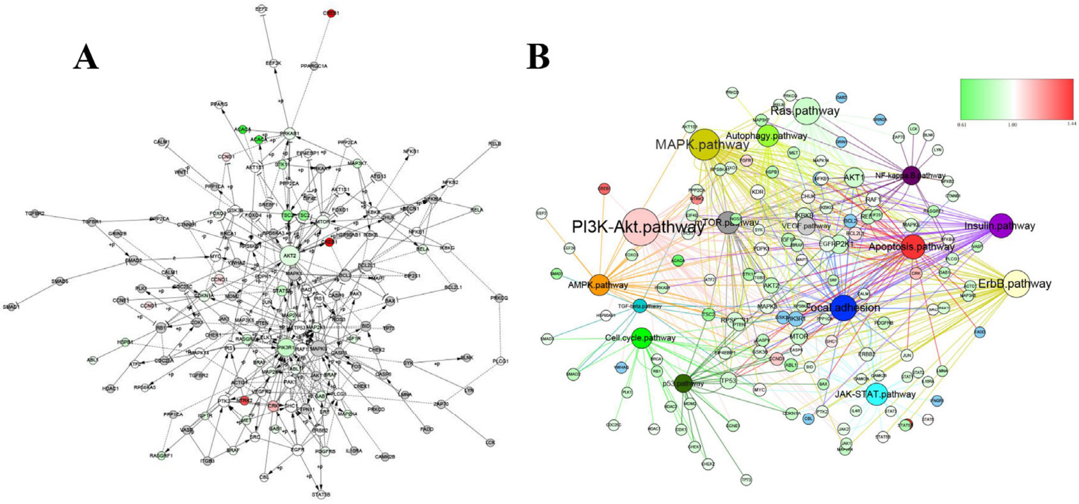
A
B
PI3K-Akt.pathway
MAPK.pathway
Ras.pathway
Autophagy.pathway
Insulin.pathway
Apoptosis.pathway
ErbB.pathway
Focal.adhesion
JAK-STAT.pathway
AMPK.pathway
Cell.cycle.pathway
AKT1
